# Ethnomycological Investigation in Serbia: Astonishing Realm of Mycomedicines and Mycofood

**DOI:** 10.3390/jof7050349

**Published:** 2021-04-29

**Authors:** Jelena Živković, Marija Ivanov, Dejan Stojković, Jasmina Glamočlija

**Affiliations:** 1 Institute for Medicinal Plants Research “Dr Josif Pancic”, Tadeuša Košćuška 1, 11000 Belgrade, Serbia; jelenazivkovic1@yahoo.com; 2 Department of Plant Physiology, Institute for Biological Research “Siniša Stanković”—National Institute of Republic of Serbia, University of Belgrade, Bulevar despota Stefana 142, 11000 Belgrade, Serbia; marija.smiljkovic@ibiss.bg.ac.rs (M.I.); jasna@ibiss.bg.ac.rs (J.G.)

**Keywords:** ethnomycology, Balkan, food, medicines, mushrooms, macrofungi

## Abstract

This study aims to fill the gaps in ethnomycological knowledge in Serbia by identifying various fungal species that have been used due to their medicinal or nutritional properties. Ethnomycological information was gathered using semi-structured interviews with participants from different mycological associations in Serbia. A total of 62 participants were involved in this study. Eighty-five species belonging to 28 families were identified. All of the reported fungal species were pointed out as edible, and only 15 of them were declared as medicinal. The family Boletaceae was represented by the highest number of species, followed by Russulaceae, Agaricaceae and Polyporaceae. We also performed detailed analysis of the literature in order to provide scientific evidence for the recorded medicinal use of fungi in Serbia. The male participants reported a higher level of ethnomycological knowledge compared to women, whereas the highest number of used fungi species was mentioned by participants within the age group of 61–80 years. In addition to preserving ethnomycological knowledge in Serbia, this study can present a good starting point for further pharmacological investigations of fungi.

## 1. Introduction

Ethnomycology represents an area of ethnobiology that investigates traditional knowledge, as well as cultural and environmental effects, of the association between fungi and man through time and space [1]. Fungi are an important part of ecosystems, where they play many essential roles. They facilitate plants’ access to nutrients and water; serve as decay agents that cycle carbon and nutrients through the soil, water and atmosphere; and are major regulators of microbial populations [2]. It is widely known that they have been used historically and globally as food, as well as in medicine. However, ethnomycological knowledge is understudied when compared to local folkloric knowledge about animals and plants. Despite the historical use of fungi since antiquity, ethnological studies of wild mushrooms are a relatively new phenomenon [3].

Mycology has a strong and long tradition in Europe and the knowledge of European species, their distribution, ecology and status is the most extensive in the world [4]. On the other hand, ethnomycological studies have until recently been scarcely performed in Europe. Due to the fact that the usage of fungi as nutraceuticals is increasing, in the last few years an increasing interest in this type of study can be observed [5]. Recent studies have indicated that mushrooms are indeed functional foods, containing components that can contribute to human wellness and mitigate threats and assaults that render the human body vulnerable to several life-threatening diseases, including cardiovascular ailments, cancer and metabolic (diabetes and obesity) and neurodegenerative disorders [6]. Therefore, ethnomycological investigations are of prime importance for the further scientific validation of their traditional uses and possible introduction to the development of modern drugs. Furthermore, the documentation of traditional knowledge on the identification of edible wild mushrooms is vital for the application of appropriate management strategies and for the transmission of this knowledge to new generations [7].

As part of Europe, the Balkan peninsula, despite its immense biological and cultural diversity, has begun to attract the attention of ethnobiologists only in the last decades [8]. Thereafter, various studies have addressed folk knowledge in a number of fields, although there are still not many data on the use of fungi in this part of Europe. The richness of fungi and the narrowness of ethnomycological investigations in the Balkan region make it an ideal area for further research.

This study aims to fill the gaps in the ethnomycological knowledge of the Balkan Peninsula, in particular in Serbia. The primary objective was to document fungal taxa that are traditionally used in Serbia. We collected data related to the identification of various fungus species and their edibility, medicinal and food properties, as well as their modes of preparation. Detailed analysis of the literature was also performed in order to provide scientific evidence related to the recorded medicinal use of fungi in Serbia.

## 2. Methodology

### 2.1. Ethnomycological Survey

The ethnomycological survey was carried out during 2016, with participants from different mycological associations in Serbia (Mycological Society of Serbia, Mycological Society of Novi Sad, Ecological Society Studenac, Mushroom Society Šumadija, Mushroom Society Požarevac and the Association of Mushroom and Nature Lovers Vilino kolo). Data were collected randomly using semi-structured interviews. A total of 62 participants were interviewed. The age of participants was between 18 and 80, with an average value of 59 years (Table 1) and the number of male and female participants was 22 (35.5%) and 40 (64.5%), respectively.

**Figure 1:**
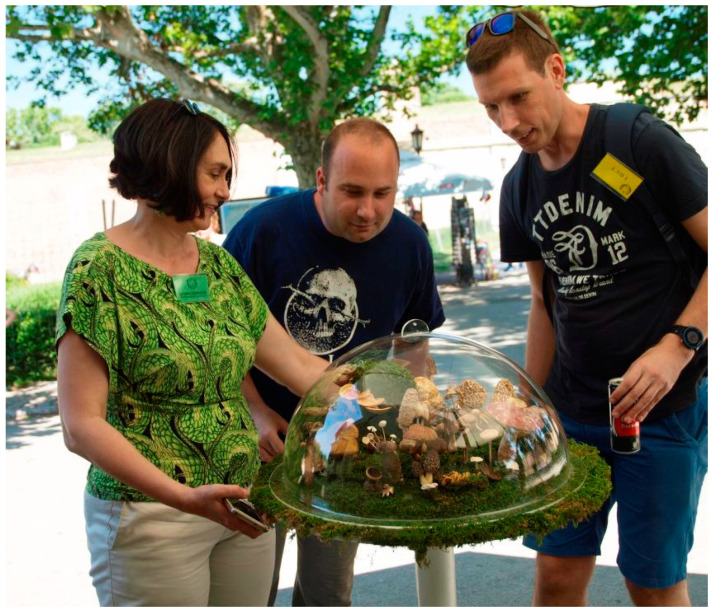
Collection of ethnomycological data in the field.

Interviews were performed orally by researchers from the Laboratory of Mycology, Department of Plant Physiology at the Institute for Biological Research “Sinisa Stankovic”. Clarifications of the context and the aim of the investigation were presented to the potential participants at the beginning of the surveys. The participants were asked to list all the fungi species they used. In particular, the interview included the following questions: respondent name, sex, age, residence, nationality, profession, local names of the fungi they use, preparation/administration and type of use. For the identification, researchers were equipped with dried fungi samples, photos and literature [9,10,11,12,13,14,15,16,17,18,19,20]. The validation of biological sample materials that were shown by participants was guided by the exact identification of species by Dr Jasmina Glamočlija, a full research professor in the mycological laboratory of the Institute for Biological Research—National Institute of Republic of Serbia, University of Belgrade, Serbia (Figure 1).

### 2.2. Data Analysis

All fungi cited by participants were taken into account for subsequent analysis. After collecting data, fungal species were ranked based on the number of times that they were mentioned by the participants. Species names and authors’ abbreviations were annotated according to the Index Fungorum site (www.indexfungorum.org; the access date 2 March 2021). The acquired data gathered were grouped in Microsoft Excel and further assessed by quantifying the use reports. Each time a fungus was indicated as “used” this was considered as one “use-report” (UR). Statistical analysis was performed using the Chi-squared test of independence, with Yates’s correction for continuity.

## 3. Results and Discussion

The results obtained during our study are presented in Table 2, in which fungi used by informants are arranged in the alphabetical order of their names. For each fungus, the scientific name and family, local names, preparation/administration, folk medical uses and total number of use reports were reported. According to our knowledge, this is the first ethnomycological study conducted on the territory of Serbia. This lack of documentation could make local mycological knowledge vulnerable.

In our study, a total of 85 species belonging to 28 families were identified. On average, each interviewed person mentioned nine edible species, with the lowest number of mentioned species at three and the largest at 50. Species with the highest number of use reports were *Pleurotus ostreatus*, *Agaricus leucotrichus*, *Boletus persoonii*, *Agaricus macrosporus*, *Macrolepiota mastoidea*, *Cantharellus cibarius* and *Leatiporus sulphureus*. The family Boletaceae was represented by the highest number of species (14), followed by Russulaceae (11), Agaricaceae (8) and Polyporaceae (8). The male participants reported the use of the higher number of fungus species compared to women. The Chi-squared test of the number of species reported by various age categories yielded significance. It was observed that the highest number of species was mentioned by participants within the age group of 61–80 years. Older generations had a good knowledge of fungi as compared to the groups below 40 years of age.

**Figure 2:**
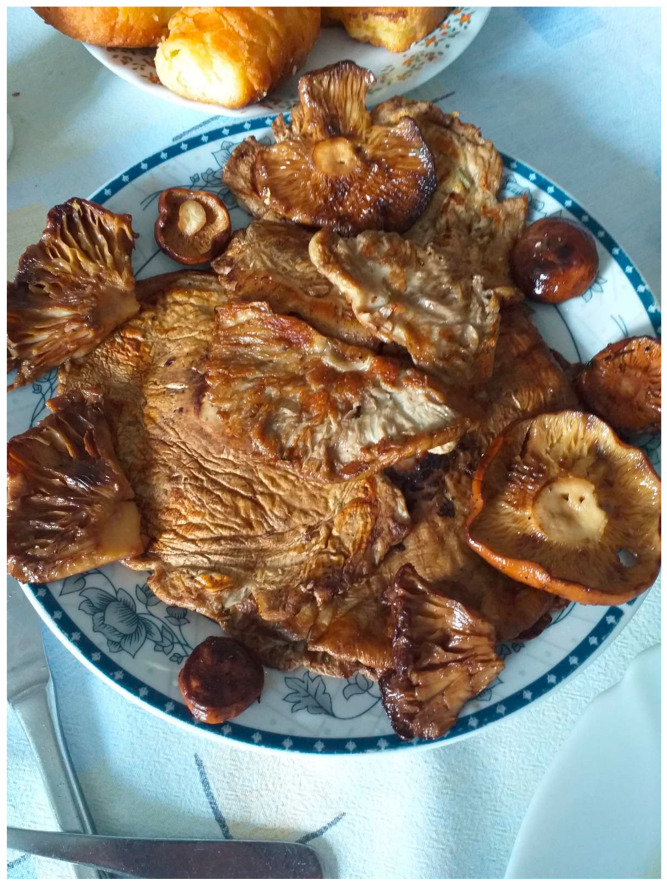
Grilled *Macrolepiota procera* and *Lactarius volemus* prepared by informants during the investigation.

With the exception of *Fomes fomentarius*, *Ganoderma applantatum*, *G. atkinsonii* and *G. lucidum*, the reported fungus species were pointed out as edible, but only 15 of them were declared as medicinal. In Serbia, fungi are commonly utilized as prominent components of traditional foods and recipes (Figure 2). According to the literature [21], they are beneficial due to their high-quality protein, fiber, mineral and vitamin contents. Moreover, their low fat content, with a high proportion of polyunsaturated fatty acids, turns them into a source of favorable fat [22]. As stated by participants, there are several methods of preparation of edible fungi. They mainly require preparation before consumption, and usually all parts of the fruiting body are used. They mentioned that fungi are consumed cooked or fried, especially during fasting, when they are consumed as a substitute for meat. In addition, it was reported that two poisonous fungi have been used in nutrition (*Tricholoma terreum* and *Verpa bohemica*). Namely, they are used cooked, although the water in which they were boiled should be removed after processing. The reason for this is the possible presence of thermolabile toxins.

Today, it is not unusual for urban people, particularly from the middle class, to collect fungi for their own consumption. Furthermore, the commercial harvesting of wild edible fungi also occurs [5]. According to our survey, almost all of the participants (84%) collected edible fungi for their households. In Serbia, both commercial and personal collections of wild mushrooms do not represent a current threat to fungal biodiversity [23]. In fact, there are much greater risks to fungal biodiversity, particularly air pollution and the use of fertilizers and pesticides.

Ethnomedicinal applications are one of the most important features of fungi, meaning that they are of nutraceutical value. Nowadays, numerous dietary supplements, in the form of extracts, tinctures and capsules, are made from medicinal fungi [24]. These are utilized for the treatment of various health conditions, including modern lifestyle diseases, as well as for their prevention. Regardless of the longstanding tradition of the utilization mushroom preparations in the therapy of human diseases, scientific confirmations of the effects of these preparations are meagre and are limited to a small number of species [25]. Namely, some of these mushrooms compounds were used in clinical trials and the obtained results provided supporting data for their medicinal applications [21]. Generally, studies dealing with mushrooms as therapeutic agents are focused mainly on in vitro data.

In our study, about 30% of the participants confirmed that they used fungi as medicine, whereas 15 fungal species (18% of all recorded species) were utilized due to their positive effects on health. In the following, we evaluate the status of scientific research and application for each of these species. Most of these species were consumed internally (90%), whereas only one species (*Calvatia gigantea*) was utilized both internally and externally.

### 3.1. Auricularia auricula-judae (Bull.) Quél

According to Gründemann et al. [26], the use of this mushroom in European ethnomedicine has a very long tradition, and it is gargled with for the treatment of sore throats, as well as sore eyes and jaundice and as an astringent. The mushroom is also highly appreciated throughout the Asian continent. It is also known as “tree jellyfish”, “wood ear” or “black mushroom”, and it has long tradition of use both as food and medicine. Some data indicate that it has potential in treating throat-related ailments, as well as thrombosis deterrence. Furthermore, it is a valuable source of bioactive glucans and essential amino acids and has a fair amount of minerals (Ca, K, Mg, Fe, Zn) which are irreplaceable in the human diet [27].

The traditional use of this mushroom as an agent in blood vessel purification (Table 1) may have recently found its scientific support. According to Bian et al. [28], fruiting bodies of mushroom are a source of acidic polysaccharides with glucuronide (defined as aAAP-1), which exhibited anticoagulant activity. As confirmed by Yoon et al. [29], polysaccharides from this mushroom are those with anticoagulant activity due to catalysis of thrombin inhibition by antithrombin, with glucuronic acid being the essential factor for this polysaccharide activity.

### 3.2. Boletus edulis Bull

*Boletus edulis,* also known as the king bolete, is widely consumed throughout Europe, North America and Asia. It is especially appreciated due to its flavor—a mixture of nutty, earthy and meaty. The most abundant bioactive constituents of this mushroom are antioxidants such as ascorbic acid; tocopherols, including α-tocopherol, γ-tocopherol and δ-tocopherol; and phenolic acids [30].

Various fractions/molecules present in *B. edulis* may be the carriers of its observed immunomodulatory capacity (Table 2). The study by Lemieszek et al. [31] has linked the immunomodulatory activity of *B. edulis* to its RNA fraction. This fraction was able to stimulate NK92, a natural killer cell line, inducing their proliferation and cytotoxic potential towards tumor cells. *B. edulis* is also highlighted as a rich source of B3 vitamin [32], a molecule that stimulates the immune response against pathogenic bacteria [33]. Among the most abundant *B. edulis* constituents are phenolic compounds, including p-coumaric acid [34]; due to its antioxidant capacity, this molecule has immunomodulatory properties in both physiological and pathological states [35].

### 3.3. Calvatia gigantea (Batsch)

*Calvatia gigantea*, or giant puffball, is considered the largest among edible mushrooms and is a promising source of compounds with medicinal activity for health foods and food supplementary products [36]. In Europe these mushrooms have been traditionally used as a powder due to their hemostatic properties, as wound dressings, as well as in the treatment of inflammation [26].

In vitro studies provide evidence for the ethnopharmacological use of *C. gigantea* for the treatment of cancer (Table 2). Calvacin, a protein obtained from the spores of this fungus, was active against a range of tumors studied by Roland et al. [37]. It has been shown that this mushroom could have beneficial effects on lung cancer—the mechanism behind this cytotoxic activity is increased Bax, p53, caspase-3 and caspase-9 expression, and the downregulation of G1/S-specific cyclin D1 (CCND1), CCND2, CDK4, Akt and Bcl-2 in A549 lung cancer cells [38]. One of the most abundant molecules of *C. gigantea* is gentisic acid [36], a phenolic compound which reduces the number of HT29 cells (human colorectal adenocarcinoma cell line) [39]. On the other hand, its most abundant sugar compound, trehalose [36], inhibited cell proliferation in two melanoma cell lines (A375 and SK-Mel-28) [40]. There are records even from few hundred years ago regarding the ability of this fungus to prevent bleeding from wounds [41]. Calvacin gel, composed of *Calvatia gigantea* (spore) and *Gentiana macrophylla* (herb), increases levels of VEGF and TGFβ1 in rats, leading to wound healing [42], altogether giving scientific support to the observed usage of C. *gigantea* among the Serbian population (Table 2).

### 3.4. Cantharellus cibarius Fr

*Cantharellus cibarius,* or gold chanterelles, is among the most frequently consumed forest mushrooms in Europe due to its apricot scent and its enticing yellow or orange color [43].

*Cantharellus cibarius* usage as a disease prevention agent among Serbian inhabitants (Table 1) could be linked to its abundant bioactive molecules. The agents with antiproliferative/cytotoxic properties obtained from this mushroom are numerous and include small RNAs [44], galactan [45] and branched mannans [46]. Polysaccharides from *C. cibarius* exhibit anti-inflammatory effects by inhibiting COX-1 and COX-2 activity [47], whereas 3-*O*-methylated galactan activates macrophages [48]. Polysaccharides have also displayed beneficial effects as neuroprotection agents [49]. *Cantharellus cibarius*, in a study by Kala et al. [50], was the richest source of lovastatin among all the mushrooms, indicating its potential application in the prevention of hypercholesterolemia.

### 3.5. Coprinus comatus (O.F.Müll.) Pers

*Coprinus comatus*—shaggy ink cap, lawyer’s wig or shaggy mane—is an edible mushroom found and cultivated worldwide, and is a rich source of nutritional and bioactive compounds [51].

The anti-diabetic potential of *C. comatus* was confirmed through in vitro experiments (Table 2). A study by Stojković et al. [52] highlighted the methanol extract of this species as the most active one in the inhibition of enzymes linked to type-2 diabetes, especially α-amylase. Comatin, a chemical isolated from *Coprinus comatus* fermentation broth, exhibited hypoglycemic effects in both normal and alloxan-induced-diabetic rats in a study by Ding et al. [53]. Polysaccharide fractions obtained from this mushroom have also been shown to reduce blood glucose concentrations in 120 min and have a long-term hypoglycemic effect, possibly linked to immune stimulation [54].

### 3.6. Cyclocybe aegerita (V. Brig.) Vizzini

*Cyclocybe aegerita*, known as black poplar mushroom or chestnut mushroom, is cultivated in countries such as Italy due to its pleasant taste. It is rich in carbohydrates, ash and proteins [55].

The recorded usage of *C. aegerita* as a disease preventive agent (Table 1) is strongly supported by the range of bioactivities recorded for this mushroom extract and/or single compounds. *Cyclocybe aegerita* bioactive properties include a strong antioxident potential [55], that might be linked to the presence of antioxidant peptides [55] and polysaccharides [56]. Its antimicrobial and anti-quorum sensing traits [57] suggest its ability to be applied as infection-preventive agent. Some of *C. aegerita*’s bioactive components are galectin, with anti-metastatic activity [58]; ageritin, which displays antimicrobial as well as antiproliferative properties [59]; and serine proteases, which could serve as anticoagulant and antithrombolytic agents [60].

### 3.7. Fomitopsis betulina (Bull.) B.K.Cui, M.L.Han and Y.C.Dai

*Fomitopsis betulina*, birch polypore, is a wood-rotting medicinal and edible (when young) mushroom. It has been commonly applied in the folk medicine of Russia, Poland and other Baltic countries, primarily as an antiparasitic and antimicrobial agent and to stop wound bleeding. For these purposes, it is used orally as a tea or snuffed as a powder or ash [26]. In addition, it has been traditionally used for a range of diseases such as cancers [61]. Its use as a tea for enhancing immunity was previously recorded for *F. betulina* in Russia [61]. However, when its immunomodulating activity was evaluated in vitro by Shamtsyan et al. [62], this species did not exhibit a strong effect as compared to other evaluated Basidiomycetes. Further scientific studies should be conducted to provide evidence or rebut the traditional usage of *F. betulina* as an immune booster.

### 3.8. Ganoderma applanatum (Pers.) Pat

*Ganoderma applanatum,* artist’s bracket, is found throughout the world, mainly on the bases of tree stumps. It has been used in Chinese herbal medicine, along with *G. lucidum*, in order to treat or prevent a range of chronic diseases [63].

The exo-biopolymer from this fungus, mainly containing carbohydrates, is able to increase the activity of natural killer cells [64]. Polysaccharides from *G. applanatum* were able to regenerate NK cell activity and the IL2 and IFNγ production of the spleen cells in sarcoma 180 transplanted mice [65], altogether giving support for its observed traditional usage in the Serbian population (Table 2).

### 3.9. Ganoderma atkinsonii Jahn, Kotl. and Pouz

*Ganoderma atkinsonii* is found in Europe and in the Euro-Asian region. Some records [66] indicate its promising antioxidant potential due to the presence of bioactive molecules such as 2,5-dihydroxybenzoic acid and vanillic acid. Vanillic acid is also proven to have an immunomodulatory effect through peripheral blood mononuclear cell (PBMC) stimulation and IFN-γ secretion [67], so this might be one of the components responsible for its bioactivities.

### 3.10. Ganoderma lucidum (Curtis) P. Karst

*Ganoderma lucidum* is one of the most widely recognized medicinal mushrooms. Some of the indications for its usage are bronchitis, asthma, hypercholesterolemia, hepatopathy, hypertension, arthritis, neurasthenia, hypertension and immunological diseases, all due to numerous bioactive molecules present in this fungus—phenolic compounds, polysaccharides and terpenes [68].

Extracts from this fungus regulated the expression levels of serum immune cytokines and enhanced the anti-tumor immunostimulatory activity in a Hepa1-6-bearing C57 BL/6 mouse model [69]. The mycelium of *G. lucidum* stimulates innate immunity through the activation of NF-kappaB [70] and various studies have shown that *G. lucidum* can act on different components of immune defense, including antigen-presenting cells, NK cells and T and B lymphocytes [71]. Even a randomized, double-blind and placebo-controlled study in children with cancer confirmed the immunomodulatory potential of *G. lucidum* [72], highlighting it as a promising immune enhancer, as was observed in Serbian population (Table 2).

A bioactive mixture containing *G. lucidum* described by Lu et al. [73] exhibited great potential in oral cancer treatment. It induced cell apoptosis and inhibited migration as well as cyclin expression, and also significantly reduced tumor growth in mice. On the other hand, its observed usage in the control of high blood pressure (Table 2) was not supported by evidence from a double-blind, randomized, placebo-controlled trial conducted by Klupp et al. [74]. This study does not suggest the use of *G. lucidum* for the treatment of cardiovascular risk factors in people with diabetes mellitus or metabolic syndrome.

### 3.11. Lentinula edodes (Berk.) Peger

*Lentnula edodes*, shiitake mushroom, has been cultivated throughout Asia for many years, and according to traditional Chinese medicine it exhibits beneficial effects on heart health, lung diseases and many other indications. The spectrum of its biological activities is associated with its nutritional profile and the presence of biologically active molecules such as dietary fiber; provitamin D2; and vitamins B1, B2, B12 and niacin [75].

Its role in the prevention of high cholesterol blood levels (Table 2) could be attributed to the presence of lovastatin, a cholesterol-lowering drug, in its fruiting bodies [50] and the hypocholesterolemic molecule eritadenine [76]. Furthermore, a *β*-glucan-enriched extract of this mushroom was able to profoundly reduce the levels of cholesterol in a mouse model [77]. Previously, it has been shown that the intake of shiitake mushroom by male Wistar rats significantly reduced cholesterol concentrations in the blood [78].

### 3.12. Meripilus giganteus (Pers.) P. Karst

*Meripilus giganteus* is a tree parasite with an edible fruiting body. It is rich in carbohydrates and proteins, whereas it presents a low fat content [79].

The immunity enhancing activity recorded in this study for *M. giganteus* (Table 1) has not been experimentally confirmed to date. This recorded activity might be linked to the presence of chemical constituents [79] with confirmed immunomodulatory activity, including cinnamic [80] and p-coumaric acids [35].

### 3.13. Pleurotus ostreatus Sensu Cooke

*Pleurotus ostreatus,* oyster mushroom, is the second most cultivated edible mushroom worldwide, after *Agaricus bisporus*. It is rich in β-glucans and dietary fibers, with a range of biological roles [81].

Clinical trials have demonstrated the beneficial effects of *P. ostreatus* intake on glucose metabolism, mainly the reduction in fasting and/or 2 h postprandial glucose [81], giving support for its use in diabetes indications (Table 2). A diet enriched with *Agaricus bisponus* and *Pleurotus ostreatus* eliminated the negative effects of diabetes in experimental animals by reducing the serum glucose level after 28 days of application [82]. The anti-diabetic and anti-hyperglycemic effects of *P. ostreatus* were linked to molecules such as ergosterol [83] and polysaccharides [84].

*P. ostreatus* has been used among the studied population in order to enhance immunity (Table 2). In previous studies, food supplementation with *P. ostreatus* increased levels of interleukin-2, immunoglobulin G and immunoglobulin M; tumor necrosis factor-α; as well as immunoglobulin A in piglets [85]. The addition of aqueous extracts of *P. ostreatus* to the diet of malnourished mice enhanced their humoral immunity and activated macrophage cells [86]. The immunomodulatory effect of oyster mushrooms could be attributed to the prescence of lectin [87] and pleuran-β-glucan [88].

### 3.14. Trametes versicolor (L.) Lloyd

*Trametes versicolor*, turkey tail, is a medicinal mushroom that is widely used due to its medicinal value, with characteristic morphological features—concentric multicolored zones on the upper side of the cap [89].

Polysaccharopeptides from *T. versicolor* modulate immunity by reducing levels of TLR4, MyD88, CD14, IL-1β and TNF-α expression [90], whereas polysaccharides bind and activate B cells [91]. Glucan from this mushroom exhibits immunomodulatory activity by increasing the secretion of various interleukins and interferons [92], and *β*-glucans from *T. versicolor* can also protect mice from *Salmonella typhimurium* infection by enhancing the activity of innate immune cells [93]. The current knowledge on the immunomodulatory effects of *C. versicolor* and its components has been reviewed by Chu et al. [94], Saleh et al. [95] and Habtemariam [89], and these studies provide strong support for its traditional usage in the Serbian population (Table 2).

## 4. Conclusions

The Balkan Peninsula, which is very rich in mushroom genetic resources, deserves more intensive ethnomycological studies. The current study provides useful documentation, which can contribute to preserving ethnomycological knowledge in Serbia. Interest in mycology is on the rise, as evidenced by the high number of species used by amateur mycologists. This interest contributes to the development of more detailed knowledge on mycomedicinces and mycofood. There is a need for further ethnomycological studies in order to preserve the current knowledge, since the use of fungi as food and medicine was mainly recorded among the elderly population.

## Figures and Tables

**Table 1:** Demographic features of participants.

Age of Informants	Number of Participants
<20	1
20–40	9
41–60	19
61–80	33
>80	0

**Table 2:** Ethomycological data collected on the use of fungi as food and medicines (+ stands for recorded use; / stands for not recorded use).

Scientific Name, Family Name	Local Serbian Name	Food Use	Medicinal Use	Number of Use Reports
*Agaricus campestris* sensu Cooke 1885, Agaricaceae	rudnjača	+	/	4
*Agaricus cupreobrunneus* (Jul. Schäff. and Steer) Pilát 1951, Agaricaceae	braon šampinjoni	+	/	1
*Agaricus leucotrichus* F.H. Møller 1952, Agaricaceae	beli šampinjoni	+ Mushroom pie; fried mushrooms, stuffed mushrooms	/	41
*Agaricus macrosporus* (F.H. Møller and Jul. Schäff.) Pilát 1951, Agaricaceae	kračun, veliki šampinjon	+ Mushroom pie; fried mushrooms, stuffed mushrooms	/	32
*Amanita caesarea* (Scop.) Pers. 1801, Amanitaceae	blagva	+ Fresh in salads; bread spread prepared using fresh mushrooms, lemon juice, pepper and garlic; fried on butter	/	9
*Amanita rubescens* (Pers. ex Fr.) Gray 1797, Amanitaceae	biserke	+	/	3
*Amanita vaginata* (Bull.) Lam 1783 Amanitaceae Rose, Amanitaceae	siva preslica	+	/	1
*Armillaria mellea* sensu Massee 1871, Physalacriaceae	puze, mednjače	+ Cooked and mixed with paprika during ajvar preparation	/	9
*Armillaria ostoyae* (Romagn.) Herink 1973, Physalacriaceae	puze, mednjače	+	/	4
*Armillaria tabescens* (Scop.) Emel 1921, Physalacriaceae	grmače	+	/	2
*Auricularia auricula-judae* (Bull.) Quél. 1886, Auriculariaceae	judino uvo	+ Used fresh in salads or cooked in soups	+ Used to improve blood vessel function: mixed with garlic, parsley leaves and apple vinegar tea fresh mushrooms (2–3 pieces) should be nibbled	8
*Boletus edulis* Bull. 1782, Boletaceae	letnji vrganj	+ Used in goulash or fried in butter with pepper; in mushroom pie, for soups	+ Used for strengthening the immune system	38
*Boletus aereus* Bull 1789, Boletaceae	crni vrganj	+ Used in goulash or fried in butter with pepper; in mushroom pie, for soups	/	18
*Boletus pinophilus* Pilát and Dermek 1973, Boletaceae	borov vrganj	+ Used in goulash or fried in butter with pepper; in mushroom pie, for soups	/	10
*Boletus aestivalis* (Paulet) Fr. 1838, (*B. reticulatus*), Boletaceae	prolećni vrganj	+ Used in goulash or fried in butter with pepper; in mushroom pie, for soups	/	12
*Butyriboletus appendiculatus* Schaeff. 1774, Boletaceae	smeđi vrganj	+ Used in goulash or fried in butter with pepper; in mushroom pie	/	6
*Butyriboletus regius* (Krombh.) D. Arora and J. L. Frank 2014, Boletaceae	crveni vrganj	+ Used in goulash or fried in butter with pepper; in mushroom pie	/	7
*Calvatia gigantea* (Batsch) Lloyd 1904, Lycoperdaceae	velika puhara	+	+ Against tumors To stop bleeding (wound should be sprinkled with spores)	7
*Calvatia utriformis* (Bull.) Jaap 1918, Lycoperdaceae	kruškasta puhara	+	/	1
*Calocybe gambosa* (Fr.) Donk 1962, Lyophyllaceae	đurđevače	+	/	10
*Cantharellus cibarius* Fr. 1821, Cantharellaceae	lisičarke	+ Used fried, fresh in salads, in goulash	+ Used for preparation of medicinal rakia (in 2 L of vodka, 250 g of dried mushroom should be soaked for 30–45 days). This alcoholic drink has a pleasant aroma of apricot and should be used as preventative	31
*Coprinopsis atramentaria* (Bull.) Redhead, Vilgalys and Moncalvo 2001, Psathyrellaceae	sivi jarčić	+	/	2
*Coprinus comatus* (O.F. Müll.) Pers. 1797, Agaricaceae	gnojištarke, smetličarke, beli jarići	+ Used cooked or fried; dried and used as chips	+ Used for lowering blood sugar levels	14
*Craterellus cornucopioides* (L.) Pers. 1825, Cantharellaceae	mrka truba, crna truba	+ Mushroom risotto	/	11
*Cyclocybe aegerita* (V. Brig.) Vizzini 2014, Tubariaceae	jablanovače	+ Pie with mushrooms and stinging nettle; mushroom in aspic; goulash	+ Used for preparation of medicinal rakia (in 25 L of rakia, 1 kg of dried mushroom is soaked for 30–60 days). This alcoholic drink should be used as preventative	21
*Entoloma clypeatum* (L.) P. Kumm. 1871, Entolomataceae	šljivovače	+ Used for mushroom pie	/	5
*Fistulina hepatica* (Schaeff.) With. 1792, Fistulinaceae	jetrenjače, volovski jezik	+ Fresh in salads; used for preparation of sweet pie; pouched desert prepared from *F. hepatica*, *Leatiporus sulphureus* and *Sarcoscypha cocciena*	/	6
*Flammulina velutipes* (Curtis) Singer 1951, Tricholomataceae	zimska panjevica	+	/	4
*Fomes fomentarius* (L.) Fr. 1849, Polyporaceae	trud	/	+ As a preventative: tea (one spoon of dried and ground mushroom should be cooked in 1 L of water for 30 min)	1
*Fomitopsis betulina* (Bull.) B.K.Cui, M.L.Han and Y.C.Dai 2016, Fomitopsidaceae	brezina guba	+ Mushroom should be eaten while still young	+ As a preventative: tea (one spoon of dried and ground mushroom should be cooked in 1 L of water for 30 min) tincture (100 g of mushroom should be soaked in 1 L of brandy or vodka and left for 21 days, with daily shaking. After this period, tincture should be decanted and mushrooms further boiled in 3 L of water for 3 h. Tincture and tea should be mixed after cooling and this preparation should be drunk 1 to 3 times per day in small cups)	1
*Ganoderma applantatum* (Pers.) Pat. 1887, Polyporaceae	pljosnata sjajnica	/	+ For strengthening the immune system: tea (mushroom should be boiled in water for 10 min)	1
*Ganoderma atkinsonii* syn *Ganoderma carnosum* Pat. 1889, Polyporaceae	jelina sjajnica	/	+ For strengthening the immune system: +tea (mushroom should be boiled in water for 10 min)	1
*Ganoderma lucidum* (Curtis) P. Karst 1881, Polyporaceae	reiši, hrastova sjajnica	/	+ For strengthening the immune system; cancer treatment: tea (recipe 1)—mushroom should be boiled in water for 10–15 min, or it could be soaked in cold water for 5 min and then cooked for 30 min; this tea is also used as a base for preparation of Turkish coffee alcoholic tincture (recipe 1)—100 g of mushroom should be soaked in 1 L of brandy or vodka and left for 21 days, with daily shaking. After this period the tincture should be decanted and mushrooms further boiled in 3 L of water for 3 h. The tincture and tea should be mixed after cooling and this combined preparation drunk 1 to 3 times per day in small cups) tea (recipe 2)—chopped (fresh or dried) mushroom should be put in a dish (up to 20% of the volume), poured over with water and left overnight. In the morning, the mushroom should be cooked in the same water for 3 h (one informant mentioned that the same mushroom can be used for cooking three times). After cooling, the obtained extract should be divided in smaller amounts and left in the refrigerator. The obtained ice cubes should be further mixed with lemonade or mineral water and taken regularly. alcoholic tincture called Ganodermovaca (recipe 2)—mushroom should be chopped into tiny pieces, put in a glass jar or bottle and poured over with rakia obtained from grapes, apples or pears. After one month of standing, the extract should be decanted and the process should be repeated. This time it should stand in rakia 2–3 months. Two extracts should be mixed and taken regularly, alone or in combination with previously mentioned ice cubes.	4
*Grifola frondosa* (Dicks.) Gray 1821, Meripilaceae	zec gljiva	+	/	7
*Hericium coralloides* (Scop.) Pers. 1794, Hericiaceae	bukova brada	+	/	1
*Hygrophorus marzuolus* (Fr.) Bres. 1893, Hygrophoraceae	martovka	+	/	2
*Limacium russula* (Schaeff. ex Fr.) Ricken 1915, syn. *Hygrophorus russela*, Hygrophoraceae	medenka	+	/	1
*Hydnum repandum* L. (1753), Hydnaceae	ježevice	+ Prepared with sour dressing; deep fried	/	2
*Infundibulicybe geotropa* (Bull.) Harmaja 2003, syn *Clitocybe geotropa* Tricholomataceae	martinčica	+	/	1
*Lactarius deliciosus* (L.) Gray 1821, Russulaceae	rujnice	+ Cooked and mixed with paprika during ajvar preparation; salad prepared with boiled mushrooms, vinegar and garlic	/	5
*Lactarius deterrimus* Gröger 1968, Russulaceae	smrekina mlečnica	+ Deep fried or grilled	/	3
*Lactarius volemus* (Fr.) Fr. 1838, Russulaceae	presna mlečnica	+ Deep fried or grilled	/	3
*Lactarius pergamenus* syn Lactiflus glaucescens (Crossl.) Verbeken 2012, Russulaceae	mlečnica	+ Deep fried or grilled	/	1
*Lactarius piperatus* (L.) Pers. 1797, Russulaceae	paprenjače, bele ljute,	+ Deep fried or grilled	/	1
*Lactarius salmonicolor* R. Heim and Leclair 1953, Russulaceae	jelova mlečnica	+ Deep fried or grilled	/	2
*Lactarius semisanguifluus* R. Heim and Leclair 1950, Russulaceae	jelina rujnica	+ Deep fried or grilled	/	1
*Leatiporus sulphureus* (Bull.) Murrill 1920, Polyporaceae	šumsko pile	+ Used in goulash; breaded and fried; poached desert prepared from *Fistulina hepatica*, *L. sulphureus* and *Sarcoscypha coccinea.* One informant suggested the use of mushrooms grown on wild cherry trees due to their special taste and intensive color. Furthermore, only young and soft mushrooms should be used.	/	29
*Leccinum aurantiacum* (Bull.) Gray 1821, Boletaceae	crveni dedovi	+	/	3
*Leccinellum crocipodium* (Letell.) Bresinsky and Manfr. Binder 2003, Boletaceae	žuti djed	+	/	1
*Leccinium duriusculum* (Schulzer ex Kalchbr.) Singer 1947, Boletaceae	topolovi dedovi	+	/	1
*Leccinium griseum* (Quél.) Singer 1966, Boletaceae	grabovi dedovi	+	/	1
*Leccinium albostipitatum* den Bakker and Noordel. 2005, Boletaceae	belonogi dedovi	+	/	1
*Leccinium versipelle* (Fr. and Hök) Snell 1944, Boletaceae	brezovi dedovi	+	/	1
*Lentinula edodes* (Berk.) Peger 1976, syn *Tricholoma shiitake* (J. Schröt.) Lloyd 1918, Omphalotaceae	šitake	+ Cooked (in soups) and fried	+ For lowering cholesterol and trygliceride levels	4
*Lentinus tigrinus* (Bull.) Fr. 1825, Polyporaceae	tigrice-vrbovače	+	/	1
*Lycoperdon perlatum* Pers. 1796, Lycoperdaceae	tikvasta puhara	+ Breaded and fried	/	2
*Lycoperdon pyriforme* Bull. 1792, Lycoperdaceae	kruškasta puhara	+ Breaded and fried	/	1
*Macrolepiota mastoidea* (Fr.) Singer 1951, Agaricaceae	sisasta sunčanica	+ Used as a pizza base; in goulash, breaded and fried or grilled	/	11
*Macrolepiota procera* (Scop.) Singer 1948, Agaricaceae	sunčanice	+ Used as a pizza base; in goulash; breaded and fried or grilled, dried and pulverized as a spice for soups	/	32
*Macrolepiota rhacodes* syn Chlorophyllum rhacodes (Vitt.) Singer 1951, Agaricaceae	čupava sunčanica	+	/	5
*Marasmius oreades* (Bolton) Fr. 1836, Marasmiaceae	vilin klinčić	+	/	1
*Meripilus giganteus* (Pers.) P. Karst. 1882, Meripilaceae	jastrebača	+	+ For strengthening the immune system, tea is prepared	1
*Morchella conica* Krombh. 1834, Morchellaceae	kupasti smrčak	+ Stuffed mushroom; in soups, mushroom in aspic; mushroom pie	/	14
*Morchella esculenta* var *crasipes* (Vent.) Bresinsky and Stangl 1962, Morchellaceae	debelonogi smrcak	+ Stuffed mushroom; in soups; mushroom pie	/	8
*M. esculenta* var. *umbrina* (Boud.) S. Imai 1954, Morchellaceae	tamni smrcak			
*M. esculenta* var. *rotunda* (Pers.) Sacc. 1889, Morchellaceae	okrugli smrcak			
*M. esculenta ß vulgaris* Pers. 1801, Morchellaceae	obicni smrcak			
*Morchella steppicola* Zerova 1941, Morchellaceae	stepski smrcak	+ Fried with eggs	/	1
*Phallus impudicus* L. 1753, Phallaceae	smrdljivi pevac	+	/	1
*Pleurotus cornucopioides* (L.) Gillet 1876, syn *Craterellus cornucopioides* Hydnaceae	brestovača	+	/	2
*Pleurotus ostreatus* sensu Cooke 1883, Pleurotaceae	bukovače	+ Cooked (for soups) and fried; as preserved sour mushrooms	+ Regulation of blood glucose levels For strengthening the immune system	41
*Pleurotus sapidus* Sacc. 1887, Pleurotaceae	brestovača	+	/	1
*Polyporus squamosus* (Huds.) Fr 1821, Polyporaceae	škripavac	+	/	9
*Russula aurata* Fr. 1838, Russulaceae	zlatna rusula	+ Fried mushrooms	/	6
*Russula vesca* Fr. 1836, Russulaceae	krasnice	+ Fried mushrooms	/	8
*Russula cyanoxantha* (Schaeff.) Fr. 1863, Russulaceae	zeke	+ Shortly fried	/	6
*Russula virescens* (Schaeff.) Fr. 1836, Russulaceae	golubače	+ Fried mushrooms; pie with leek and mushrooms	/	2
*Sarcoscypha coccinea* (Gray) Boud. 1907, Sarcoscyphaceae	babino uvo	+ Poached desert prepared from *Fistulina hepatica*, *Leatiporus sulphureus* and *S. cocciena*	/	1
*Suillus granulatus* (L.) Roussel 1796, Suillaceae	borovnjača	+ With onion used for pie filling	/	3
*Trametes versicolor* (L.) Loyd 1921, Polyporaceae	ćuranov rep	/	For strengthening the immune system: tea (mushroom should be soaked in cold water and cooked for 30 min; or soaked in boiled water and left to stand in it for 15 min) tincture (mushrooms should be soaked in ethanol for two weeks)	2
*Tricholoma terreum* (Schaeff.) P. Kumm. 1871, Tricholomataceae	miška	+ Eaten 1–2 times per year, not more than 200 g. Mushroom should be boiled in water, and after the removal of water it can be used for omelet preparation; used for mushroom pie	/	1
*Tuber aestivum* Vittad. 1831, Tuberaceae	letnji tartuf	+	/	1
*Tuber melanosporum* Vittad. 1831, Tuberaceae	crni tartuf	+	/	3
*Tuber macrosporum* Vittad. 1831, Tuberaceae	tartuf	+	/	2
*Tuber brumale* Vittad. 1831, Tuberaceae	zimski tartuf	+	/	1
*Xerocomus chrysenteron* (Bull.) Quél. 1888, Boletaceae	zlatača	+	/	2
*Xerocomus subtomentosus* L. 1753 syn *Boletus subtomentosus*, Boletaceae	velika podstavka	+	/	2
*Verpa bohemica* (Krombh.) J. Schrot. 1893, Morchellaceae	češka smrčkovica	+ As an additional ingredient for the preparation of dishes due to its calamari-like taste. During boiling, the evaporations should not be inhaled, and water should be removed after cooking.	/	1

## Data Availability

The data presented in this study are available on request from the first author.
